# Benzene Exposure Near the U.S. Permissible Limit Is Associated with Sperm Aneuploidy

**DOI:** 10.1289/ehp.0901531

**Published:** 2010-01-06

**Authors:** Caihong Xing, Francesco Marchetti, Guilan Li, Rosana H. Weldon, Elaine Kurtovich, Suzanne Young, Thomas E. Schmid, Luoping Zhang, Stephen Rappaport, Suramya Waidyanatha, Andrew J. Wyrobek, Brenda Eskenazi

**Affiliations:** 1 Life Sciences Division, Lawrence Berkeley National Laboratory, Berkeley, California, USA; 2 National Institute of Occupational Health and Poison Control, Chinese Center for Disease Control and Prevention, Beijing, China; 3 School of Public Health, University of California, Berkeley, California, USA; 4 University of North Carolina, Chapel Hill, North Carolina, USA

**Keywords:** aneuploidy, benzene, chromosome X, chromosome Y, chromosome 21, fluorescent in situ hybridization, germ cells, muconic acid

## Abstract

**Background:**

Benzene is a common industrial chemical known to induce leukemia and other blood disorders, as well as aneuploidy, in both human blood cells and sperm at exposures > 10 ppm. Recent reports have identified health effects at exposure levels < 1 ppm, the permissible exposure limit (PEL; 8 hr) set by the U.S. Occupational Safety and Health Administration.

**Objective:**

We investigated whether occupational exposures to benzene near 1 ppm induce aneuploidy in sperm.

**Methods:**

We used multicolor fluorescence *in situ* hybridization to measure the incidence of sperm with numerical abnormalities of chromosomes X, Y, and 21 among 33 benzene-exposed men and 33 unexposed men from Chinese factories. Individual exposures were assessed using personal air monitoring and urinary concentrations of benzene and *trans,trans*-muconic acid (E,E*-*MA). Air benzene concentrations were not detectable in unexposed men; in exposed men, concentrations ranged from below the detection limit to 24 ppm (median, 2.9 ppm), with 27% of exposed men (*n* = 9) having concentrations of ≤ 1 ppm. Exposed men were categorized into low and high groups based on urinary E,E-MA (median concentrations of 1.9 and 14.4 mg/L, respectively; median air benzene of 1 and 7.7 ppm, respectively), and aneuploidy frequencies were compared with those of unexposed men.

**Results:**

Sperm aneuploidy increased across low- and high-exposed groups for disomy X [incidence rate ratio (IRR) = 2.0; 95% confidence interval (CI), 1.1–3.4; and IRR = 2.8; 95% CI, 1.5–4.9, respectively], and for overall hyperhaploidy for the three chromosomes investigated (IRR = 1.6; 95% CI, 1.0–2.4; and IRR = 2.3; 95% CI, 1.5–3.6, respectively). We also found elevated disomy X and hyperhaploidy in the nine men exposed to ≤ 1 ppm benzene compared with unexposed men (IRR = 1.8; 95% CI, 1.1–3.0; and IRR = 2.0; 95% CI, 1.1–3.9, respectively).

**Conclusions:**

Benzene appeared to increase the frequencies of aneuploid sperm for chromosomes associated with chromosomal abnormality syndromes in human offspring, even in men whose air benzene exposure was at or below the U.S. permissible exposure limit.

Benzene is a common industrial chemical and ubiquitous environmental pollutant, and exposure to benzene is practically unavoidable for the general population. It is present in gasoline, paints, adhesives, and solvents and is a product of gasoline combustion (Zhang et al. 2008) and cigarette smoke (Wallace et al. 1987). The U.S. Occupational Safety and Health Administration (OSHA) has set a permissible exposure limit (PEL) of 1 ppm [8-hr time-weighted average (TWA)]. Occupational benzene exposure is higher in many countries, such as China, where the national occupational exposure limit of 6 mg/m^3^ (1.9 ppm) is nearly twice that of the United States. However, recent studies indicate that workers in some Chinese factories experience exposures that exceed this limit (Liang et al. 2005; Liu et al. 2003; Wang et al. 2006).

Benzene is an established human leukemogen (International Agency for Research on Cancer 1987), and exposure has been associated with various blood disorders (Smith 1996). Increases in chromosomal aberrations in peripheral blood lymphocytes have been associated with increased risk of hematologic and other cancers (Zhang et al. 2002). Aneuploidy and chromosomal rearrangements that are frequently associated with leukemias and lymphomas have been detected in humans exposed to benzene (Zhang et al. 2002, 2005, 2007). Increases in monosomy 5 and 7; trisomy 1, 7, 8, and 21; and aneuploidy of chromosome X and t(8, 21) have been reported in lymphocytes of workers exposed to benzene at mean air concentrations of approximately 30–45 ppm compared with unexposed groups (Smith et al. 1998; Zhang et al. 1998, 2002, 2005). Of concern is that low-dose occupational exposures at concentrations < 1 ppm have been associated with hematotoxic defects such as reduced white blood cell and platelet counts (Lan et al. 2004) but were not associated with elevated aneuploidy of chromosomes 1, 7, 9, 11, 18, and X in lymphocytes (Carere et al. 1998a, 1998b; Zhang et al. 2002).

Aneuploidy and structural chromosomal abnormalities transmitted via sperm can be detrimental to the viability, development, and health of human embryos and offspring (Hassold and Hunt 2001; Wyrobek et al. 2005a). Autosomal aneuploidies in offspring are primarily due to chromosomal segregation errors during the first meiotic division of oogenesis, with only a minor paternal contribution (Hassold et al. 2007). However, aneuploidies of the sex chromosomes have a strong paternal contribution (Baumgartner et al. 1999; Eskenazi et al. 2002). About 55% of the sex-chromosomal aneuploidies, which result in Klinefelter and Turner Syndromes as well as Triple X and X-Y-Y aneuploidies, are of paternal origin (Hall et al. 2006).

Prior studies reported associations between high-dose benzene exposure (mean concentrations ranging from 13 to 27 ppm; 8-hr TWAs ranging from 42 to 86 mg/m^3^) and increased frequencies of sperm with disomy for chromosomes X, 7, 8, 9, or 18 (Li et al. 2001; Liu et al. 2000; Zhao et al. 2004) as well as sperm with chromosomal aberrations such as duplication and deletion of the centromere and telomeric regions of chromosome 1 (Liu et al. 2003).

The objectives of our study were to investigate whether men occupationally exposed to benzene at concentrations near the U.S. PEL have higher frequencies of sperm aneuploidy than unexposed men and to determine whether this relationship is dose related. We employed multicolor sperm fluorescence *in situ* hybridization (FISH) to examine aneuploidy of three chromosomes (21, X, and Y) that are compatible with viable offspring.

## Materials and Methods

### Study population and design

Benzene-exposed men were recruited from three factories in Tianjin, China, that used benzene-containing glues in the manufacture of shoes, paper bags, and sandpaper. Unexposed participants were recruited from Tianjin factories with no history of benzene use—a meat-packing plant and an ice cream manufacturing factory. Factory directors and local health authorities gave permission to conduct the study within the factories. Protocols, questionnaires, and consent forms were reviewed and approved by the Committees for the Protection of Human Subjects at the University of California, Berkeley, Lawrence Livermore National Laboratory, Lawrence Berkeley National Laboratory, and the Tianjin Occupational Disease Hospital (Tianjin 3rd Municipal Hospital) under an institutional review board authorization agreement with the National Institute of Occupational Health and Poison Control, Chinese Center for Disease Control and Prevention. Study materials were developed in English, translated to Mandarin, and back-translated.

Men were eligible for participation if they were 18–50 years of age, worked at the factory for at least 1 year, and had no history of cancer or vasectomy. One investigator (G.L.) approached workers at their job site and administered a brief screening questionnaire to assess eligibility. Men who were eligible and willing to participate were escorted to a private room at the factory where they completed the screening interview, and written informed consent was obtained for the exposure assessment phase of the study.

Ninety-six men wore a personal passive-air badge monitor (3M Organic Vapor Monitor, model 3500; 3M, St. Paul, MN, USA) for a full 8-hr workday and provided a spot urine sample at the end of the work shift. Approximately 1 month later, men provided a second air sample and spot urine sample. Men who participated in the exposure assessment phase of the study were asked if they were interested in participating in the semen phase of the study. Those who were at work on the second day of sampling and who agreed (85 men; 35 exposed and 50 unexposed) were scheduled to visit the Tianjin 3rd Hospital and were instructed to avoid ejaculation for at least 2 days prior to their appointment. At the hospital, men were interviewed and examined by a Chinese urologist; a fasting blood sample was collected by venipuncture, and men provided a semen specimen by masturbation. Seventy-eight men (34 exposed and 44 unexposed) provided an adequate semen sample of at least 1.5 mL. These semen samples were collected 3.7 ± 2.2 days (mean ± SD) after the second urine collection. We determined sperm aneuploidy for a subgroup of 34 unexposed men who were frequency-matched to the 34 exposed men on age and smoking habits.

### Exposure assessment

Passive-air monitors were individually sealed and transported at room temperature to the Chinese Center for Disease Control in Beijing, where they were stored at 4ºC prior to analysis. Analysis was performed according to the 3M Organic Vapor Method (3M 2002). Air monitors were desorbed for 30 min in 1.5 mL carbon disulfide and analyzed for benzene, toluene, and xylene by gas chromatography with flame ionization detection.

Urine samples were aliquoted within 20 min of collection and placed on dry ice for transport to the Tianjin 3rd Hospital, where they were kept at −20°C until transferred to a −80°C freezer in Beijing. Urine specimens were then shipped on dry ice to the University of North Carolina, Chapel Hill, for analyses using established methods with slight modifications (Waidyanatha et al. 2001, 2004). For urinary benzene analyses, room temperature urine samples (0.5 mL) were transferred to vials containing NaCl and [^2^H_6_]benzene as an internal standard. Samples were allowed to reach equilibrium for 30 min. Benzene was extracted by head space solid-phase microextraction using a Varian Model 8200 autosampler (Varian, Walnut Creek, CA, USA), followed by analysis by gas chromatography-mass spectrometry (Waidyanatha et al. 2001). For *trans,trans*-muconic acid (E,E-MA analyses), 0.5 mL urine was added to a mixture of internal standards including [^13^C_2_]E,E-MA. Urine samples were digested with concentrated hydrochloric acid followed by extraction with ethyl acetate. The organic layer, containing E,E-MA, was evaporated to dryness, converted to trimethylsilyl derivatives, and analyzed by gas chromatography-electron ionization-mass spectrometry (Waidyanatha et al. 2004). Appropriate quality control procedures were in place for all assays, and the limits of detection (LODs) were 0.2 ppm for air benzene, 0.016 μg/L for urinary benzene, and 10 μg/L for E,E-MA. Urinary benzene analyses were performed on all specimens from the unexposed and exposed subjects (two samples per subject), and E,E-MA analyses were performed for both samples of the exposed subjects only. Laboratories that performed air and urine analyses were blind to the origin of the samples.

### X-Y-21 sperm FISH assay

Multicolor sperm FISH was employed to determine the frequency of sperm aneuploidy for chromosomes X, Y, or 21 (Baumgartner et al. 1999; Frias et al. 2003). Aliquots of frozen semen (−80ºC) were thawed to room temperature and 5 μL was smeared onto glass microscope slides. Slides were air dried and stored under nitrogen at −20°C until hybridized. Sperm chromatin was decondensed using the DTT/LIS (dithiothreitol/ lithium 3,5-diiodosalicylic acid) method (Wyrobek et al. 1994). Three chromosome- specific probes were used: *a*) a CEP X probe (Vysis Inc., Abbott Molecular Inc., Des Plaines, IL, USA) for the X chromosome labeled with both SpectrumGreen and SpectrumOrange; *b*) a centromeric alpha satellite DNA probe for chromosome Y (Vysis) labeled with SpectrumGreen; and *c*) an LSI probe for the q-arm of chromosome 21 (Vysis) labeled with SpectrumOrange. Hybridization with these probe mixtures and posthybridization washes were performed using an established protocol (Baumgartner et al. 1999). Slides were scored using a Zeiss Axioplan fluorescence microscope equipped with a triple-band-pass filter for FITC/Texas Red/DAPI (61002; Chroma Technology Corp., Bellows Falls, VT, USA). A single scorer analyzed all samples in this study. The scorer was blind to exposure status and trained by an experienced researcher using historic semen samples with extensive scoring data. Slides were randomized and encoded by a second person (not the scorer) for scoring by the following procedure: *a*) 5,000 sperm were scored in a specified region of the hybridization area using strict scoring criteria (Baumgartner et al. 1999); *b*) every slide was recoded; and *c*) an additional 5,000 sperm were scored on a separate area of the same slide by the same scorer. The two data sets for each slide were accepted if counts for total hyperhaploidy, total hypohaploidy, and total abnormalities did not differ according to chi-square analyses. In this study, only one slide failed to meet this criterion, and a new slide was prepared and re-scored. Disomy X, Y, and 21, XY sperm, sex-null sperm, chromosome 21-null sperm, and various forms of sperm diploidy were measured separately as previously described (Baumgartner et al. 1999). Semen samples from two donors (one unexposed and one exposed) could not be analyzed because of poor hybridization quality due to high concentrations of bacteria or low sperm density.

### Statistical analysis

All statistical analyses were performed using Stata 10 for Windows (StataCorp 2007). Results from an individual’s two urine samples and personal air measurements were highly correlated (Spearman ρ = 0.9 for air benzene, 0.8 for urinary benzene, and 0.8 for urinary E,E-MA) with high intraclass correlation coefficients (0.85 for air benzene, 0.80 for urinary benzene, 0.73 for urinary E,E-MA). Exposure concentration values for air benzene, urinary benzene, and urinary E,E-MA were calculated as a summary of the geometric means (GMs) from the two collections and presented using the GM and geometric standard deviation (GSD) in addition to percentiles. Relationships between the different benzene measurements were calculated using Spearman correlations. The GM and GSD of air concentrations of benzene were not reported for unexposed men because all were < LOD. Two men in the low-exposed group also had air benzene values that were < LOD. These values were imputed as the LOD divided by the square root of 2. Categories of benzene exposure were constructed for multivariate regression models using E,E-MA concentrations because E,E-MA has been shown to be a robust biomarker of benzene exposure (Kim et al. 2006; Qu et al. 2000). Among exposed participants, concentrations of E,E-MA (summarized from the two collections) were divided at the median (6.7 mg/L). Those at or below the median were assigned to the low-exposed group, whereas those above the median were assigned to the high-exposed group.

Sperm aneuploidy was measured as the frequency per 10,000 sperm. The following categories of sperm aneuploidy were included as dependent variables: disomy X (sperm FISH genotype X-X-21); disomy Y (Y-Y-21); disomy XY (X-Y-21); disomy 21 (X-21-21 or Y-21-21); overall hyperhaploidy involving chromosomes X, Y, and 21 (sum of XY, disomy X, disomy Y, and disomy 21); sex nullisomy (_21); 21 nullisomy (X_ or Y_); overall hypohaploidy involving chromosomes X, Y, and 21 (sum of sex nullisomy and 21 nullisomy); and diploidy. “Other” was defined as all anomalies not detailed above, including sperm with multiple anomalies such as X-X_. We selected several potential confounders based on their relationships with sperm aneuploidy, semen quality, or benzene exposure in the literature: age; abstinence (days); body mass index (BMI); smoking or alcohol use in the last 3 months; fruit and vegetable intake [< median (3.6 times/day) vs. > median]; meat consumption, vitamin use (yes/no), consumption of tea and cola (yes/no); hours per day on a bicycle; number of hot baths taken per month; education (< high school vs. ≥ high school); and history of chronic disease. Participants were categorized as having a history of chronic disease if they reported having been diagnosed with any of the following conditions: tuberculosis, lung disease, anemia, diabetes, thyroid diseases, other hormonal diseases, stomach ulcers or other diseases of the gastrointestinal tract, hepatitis, liver disease, epilepsy or other neurological disorders, high blood pressure, or other diseases of the heart, blood vessels, or blood. *t*-tests, Fisher’s exact, and chi-square tests were used to assess differences between unexposed and exposed groups for potential confounders.

We used negative binomial models to assess differences in aneuploidy frequencies by exposure categories. Models were constructed for each aneuploidy outcome separately comparing the low-exposed group and high-exposed group to the unexposed group. Covariates were included in the models if they were associated with exposure and with the outcomes at *p* ≤ 0.1 in separate bivariate models or if the coefficients changed by > 10% upon removing the covariate. Although the groups were frequency-matched on age and smoking in the past 3 months, these variables were included in the models to control for any residual confounding. To simplify the analyses and the interpretation of the data, the set of covariates that met the above criteria for the majority of the outcomes was used in all models. These included age (continuous), smoking or taking hot baths in the past 3 months (yes/no), regular tea drinking (yes/no), eating fruits or vegetables > 3.6 times/day vs. ≤ 3.6 times/day, and history of any chronic disease (yes/no). Abstinence did not meet the criteria for inclusion in the models, and results did not differ whether abstinence was included or excluded from the models. Coefficients from the negative binomial models were exponentiated to give incidence-rate ratios (IRRs) comparing the high-exposed and low-exposed groups with the unexposed group. We also performed a test for trend using an independent variable that was coded as 0 for unexposed, 1 for low-exposed, and 2 for high-exposed men in separate adjusted negative binomial models. Zero-inflated negative binomial models produced similar results for outcomes with low detection frequencies, and the Vuong test indicated that standard negative binomial models were equally preferable.

## Results

Table 1 shows the characteristics of our population of exposed and unexposed workers. Participants were matched for age and smoking history and therefore did not differ in these characteristics. The mean age (± SD) for the exposed and unexposed men was 32 ± 8 years (range, 19–45 years for exposed and 19–49 years for unexposed), and mean daily cigarette use was 9 ± 10 cigarettes/day (range: 0–40 cigarettes/day for exposed vs. 0–25 cigarettes/day for unexposed). Most men in both groups smoked (> 70%) and drank alcohol (> 80%) during the 3 months before semen collection. Very few men in either group took vitamins (≤ 6%). Men in the unexposed group reported a longer period of abstinence prior to semen collection compared with the exposed group (mean ± SD, 10 ± 17 vs. 7 ± 5 days; *p =* 0.2; range, 2–100 vs. 2–30 days), had higher rates of chronic disease (33% vs. 12%; *p =* 0.04), were somewhat less likely to drink tea regularly (*p =* 0.07), and consumed fewer fruits and vegetables (*p =* 0.08). Men in the exposed group were less educated (only 15% had completed high school, compared with 48% in the unexposed group; *p =* 0.004) and took more hot baths in the 3 months before semen collection compared with unexposed men (64% vs. 36%; *p* = 0.03). Only two men reported having been told by a doctor that they had fertility problems; one of these men subsequently fathered a child, whereas the other man did not report fathering any children. This man was in the unexposed group.

Table 2 shows summary statistics of the three measures of exposure (passive-air badge monitor, urinary benzene, and urinary E,E-MA). These measures of exposure were highly correlated among exposed men (Spearman ρ > 0.75; *p* < 0.001 for each pair). The median concentration of urinary E,E-MA was used to divide the 33 men of the exposed group into subgroups of 17 men with low exposure and 16 with high exposure (median E,E-MA, 1.9 and 14.4 mg/L, respectively). Comparison of urinary benzene and air measures confirmed these categories. For passive-air badge measurements, benzene was not detectable (< 0.2 ppm) among unexposed men, and median concentrations for low-exposed and high-exposed men were 1.0 and 7.7 ppm, respectively. For urinary benzene, the median concentration was 0.1 μg/L among unexposed men, 4.3 μg/L among low-exposed men, and 52.5 μg/L among high-exposed men.

We analyzed 331,900, 170,934, and 160,935 sperm by FISH in the unexposed, low-exposed, and high-exposed groups, respectively. Table 3 shows the median, mean, and range of frequencies of abnormal sperm in our population. The distributions of aneuploidy frequencies were skewed to the right, with a higher mean than median for most subcategories of aneuploidy. This is because some anomalies, such as disomy or nullisomy 21, are rare events that occur only in a subgroup. For example, < 20% of men had at least one sperm with nullisomy 21 among Y-bearing sperm (Table 3, percent with anomaly).

We applied adjusted negative binomial regression models to compare the sperm aneuploidy outcomes of exposed men to unexposed men. Rates of overall hyperhaploidy, hypohaploidy, disomy X, disomy Y, and other anomalies were significantly higher among exposed men than unexposed men (data not shown). As shown in Table 4, compared with unexposed men, the incidence rate of hyperhaploidy was 1.6 times higher for men in the low-exposed group (*p* = 0.03) and 2.3 times higher for men in the high-exposed group (*p* < 0.001) after adjusting for age, smoking, hot baths, tea drinking, fruit and vegetable intake, and history of chronic disease (*p*_trend_ across three exposure groups, < 0.001). This finding was driven by the strong association between benzene exposure and disomy X and to a lesser extent by disomy Y. Low-exposed men had a 2 times higher incidence rate of disomy X sperm and high-exposed men had a 2.8 times higher incidence rate than unexposed men (*p* = 0.02 and < 0.001, respectively; *p*_trend_ = 0.001). High-exposed men also had a 2.6 times higher rate of disomy Y sperm (*p* < 0.001) than unexposed men, whereas low-exposed men did not differ from unexposed men (IRR = 1.1; *p =* 0.78; *p*_trend_ = 0.002). When we compared only the men who were exposed to ≤ 1 ppm of air benzene (*n* = 9) with unexposed men, we also observed elevated rates of hyperhaploidy [IRR (95% CI), 1.8 (1.1–3.0)] and disomy X [2.0 (1.1–3.9)]. Adjusted models also showed a strong association between benzene exposure and chromosome 21 nullisomy among Y-bearing sperm, but this may be a spurious finding due to the low number of men with this sperm anomaly (< 20% per exposure group).

**Figure 1:**
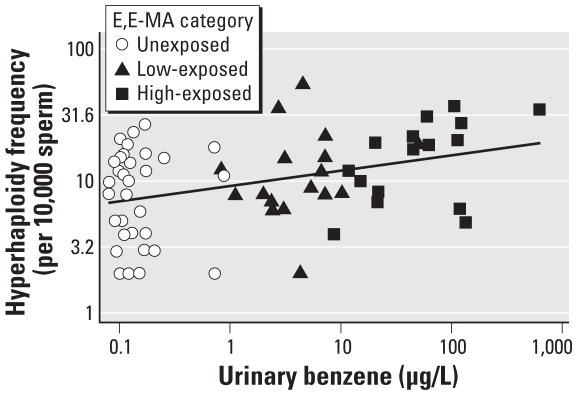
The log_10_ frequency of hyperhaploidy per 10,000 sperm increases with log_10_ urinary benzene (μg/L). β = 0.12; *p* = 0.02.

Figure 1 illustrates the dose–response relationship between log_10_ urinary benzene and log_10_ frequency of hyperhaploid sperm per 10,000 sperm with the fitted line from a linear regression (β = 0.12; *p =* 0.002) for all participants. We used urinary benzene measurements instead of E,E-MA because E,E-MA was not measured in the urine samples of the unexposed subjects. The association between urinary benzene and sperm aneuploidy remained significant even upon removal of the most extreme points of urinary benzene concentration and/or frequency of hyperhaploidy (β = 0.11; *p* = 0.002) and even when excluding unexposed men (β = 0.16; *p =* 0.05), further confirming the dose-related increase in sperm hyperhaploidy.

## Discussion

Results of the present study show that occupational exposures to benzene were associated with increased frequencies of aneuploid sperm for chromosomes X, Y, or 21. Specifically, we found significant exposure-dependent increases in the frequencies of sperm with disomy X, disomy Y, and hyperhaploidy in exposed men. Men in the low-exposed (median air benzene = 1 ppm) and high-exposed (median air benzene = 7.6 ppm) groups were 1.6 times and 2.3 times, respectively, more likely to have hyperhaploid sperm than unexposed men. Even the nine men from the low-exposed group who were exposed to ≤ 1 ppm air benzene had statistically significantly elevated rates of hyperhaploidy, specifically disomy X, compared with unexposed men. Our findings suggest that men occupationally exposed to benzene at air concentrations near the OSHA PEL of 1 ppm produce higher frequencies of aneuploid sperm for the sex chromosomes, and perhaps chromosome 21, than men who were not exposed.

The risk of abnormal reproductive outcomes of paternal origin may be influenced by male reproductive physiology and genetic factors (Hassold and Hunt 2001), past and current male environmental exposures (Olshan and van Wijngaarden 2003), or random mutational errors during sperm production (Crow 2000). Sperm FISH assays have been increasingly employed to identify factors that increase the frequencies of sperm with chromosomal abnormalities (Wyrobek et al. 2005a). Elevated frequencies of chromosomally abnormal sperm have been reported for a variety of physiological factors, lifestyle factors, and xenobiotic exposures, including increasing age (Lowe et al. 2001; Rousseaux et al. 1998), translocations (Van Hummelen et al. 1997), smoking (Shi et al. 2001), chemotherapeutic drugs (De Mas et al. 2001; Frias et al. 2003; Wyrobek et al. 2005b), and various environmental and occupational exposures (Padungtod et al. 1999; Robbins et al. 2008; Xia et al. 2005; Xu et al. 2003). Among occupational exposures, organophosphates, acrylonitrile, and benzene have been shown to increase numerical abnormalities in sperm of exposed men (Wyrobek et al. 2005a). One study found an association between paternal occupational exposure to solvents, including benzene, and spontaneous abortion (Lindbohm et al. 1991). Studies have also shown that men with higher frequencies of aneuploid sperm may be at a higher risk of fathering an aneuploid child (Lowe et al. 2001).

Although there is substantial evidence that exposure to benzene increases chromosomal abnormalities in human lymphocytes after high-dose exposures (Smith et al. 1998; Zhang et al. 2002, 2005), less is known about the induction of chromosomal abnormalities in the sperm of benzene-exposed men. To date, four Chinese studies have been published on the effects of benzene exposure on sperm aneuploidy (Li et al. 2001; Liu et al. 2000, 2003; Zhao et al. 2004). Only one study has investigated associations between benzene exposure and sex chromosome aneuploidy (Liu et al. 2000), and none have examined associations with chromosome 21 aneuploidy. All four studies were limited to small cohorts of men (~ 15) who were exposed to high air concentrations of benzene (> 10 ppm). Our study is the first to investigate aneuploidy in sperm of workers who were exposed to benzene concentrations relevant to those who are chronically exposed to air concentrations around 1 ppm (the U.S. PEL). Our study confirms the previously published associations between high benzene exposure and increases in sex chromosome aneuploidy (Liu et al. 2000), and more importantly, it extends this association to the low-dose exposure range. In our study, nine men in the exposed group had air benzene concentrations < 1 ppm (27% of all exposed men). Two of these men had higher frequencies of hyperhaploidy (36 and 54 hyperhaploid sperm/10,000) than all of the unexposed men (highest frequency, 27 hyperhaploid sperm/10,000).

Our study provides insight into the biological target cells by which benzene causes aneuploidy in human sperm. Our use of three-color FISH allowed us to compare the frequencies of various disomic and nullisomic sperm within the same samples and to assess whether the disomy and diploidy errors occurred during meiosis I (X-Y-21 and X-Y-21-21) or meiosis II (X-X-21,Y-Y-21, X-X-21-21, Y-Y-21-21). Our results suggest that benzene may preferentially affect nondisjunction of sex chromosomes rather than chromosome 21 and that meiosis II is more sensitive than meiosis I. In support of this observation, the frequency of disomy X was highly correlated with the frequency of disomy Y (*r* = 0.44, *p =* 0.004), whereas neither of these meiosis II errors were correlated with X-Y-21 (*r* = 0.08, *p =* 0.61), a type of meiosis I error. Our data also suggest that high exposures induce both disomy X and Y, whereas it appears that in the low-exposure range, benzene is more likely to induce disomy X with no detectable effects on disomy Y. This may be due to chromosome-specific susceptibilities to toxin-induced nondisjunction.

Our findings predict that occupational benzene exposures may significantly increase the risks of pregnancies with triple X and XYY syndromes, with lower and only borderline significant predicted risks for offspring with Klinefelter syndrome (XY sperm, IRR = 1.5 and 1.8 for low-exposed and high-exposed, respectively) and Down syndrome (sperm disomy 21, IRR = 2.1 and 1.6 for low-exposed and high-exposed, respectively). Our results also lend support to the growing evidence that parental exposures to benzene may predispose an offspring to childhood leukemia (Smith 2010), particularly acute lymphoblastic leukemia (ALL). Up to 40% of children with ALL have nonrandom hyperdiploidy (> 50 chromosomes) in leukemic cells, mostly with an excess gain of chromosomes X and 21 compared with other chromosomes (Paulsson and Johansson 2009). In addition, this high hyperdiploidy has been shown to occur *in utero* (Paulsson and Johansson 2009).

Our study had some limitations in design and analysis. First, our exposure assessment consisted of monitoring workplace exposure using passive-air monitors and collecting urine samples at only two time points approximately 1 month apart. Although we lacked exposure information over the entire meiotic period (about 3 months prior to collection), exposure monitoring overlapped with the timing of the two meiotic divisions (about 35 days before semen collection) when aneuploidy would be generated. Only two men (one unexposed and one low exposed) had worked < 3 months at their jobs (58 and 59 days). In addition, the strong correlation between the two time points for each measure of exposure provides confidence that we captured the usual workplace exposure of an individual. Second, we analyzed urine samples for both urinary benzene (all men) and E,E-MA (only in exposed men). We used E,E-MA to categorize benzene-exposed workers because of the comparatively short half-life of urinary benzene (Waidyanatha et al. 2004). However, analyses showed that when we used urinary benzene to categorize low-exposed and high-exposed groups, we obtained similar results (data not shown). Third, our present study and all previously published studies of sperm aneuploidy in benzene-exposed men were conducted with Chinese cohorts, and the generalizability of our findings will need to be tested in studies of other ethnic groups and in other geographic locations. Fourth, this occupational cohort may be subject to selection biases including the healthy-worker bias, whereby the individuals who are most susceptible to health effects of benzene exposure may have developed health problems that prevented them from working in the factories from which we recruited, thus underestimating effects.

## Conclusions

We found that benzene was associated with a dose-dependent increase in disomy X, disomy Y, and hyperhaploidy in the sperm of men exposed to benzene. Our findings of increased hyperhaploidy and disomy X among our low-exposed group (with a median benzene exposure of 1 ppm), as well as among the men in the low-exposed group who had benzene exposure ≤ 1 ppm, suggest that occupational exposure to benzene, even at or below the U.S. PEL, may increase the risks of spontaneous abortions and fathering children with aneuploidy syndromes or birth defects due to paternal aneuploidy. Given these findings, the current PEL of 1 ppm may not be sufficiently low to protect men from adverse reproductive outcomes that may arise from germline aneuploidy.

## Figures and Tables

**Table 1:** Population characteristics among benzene-exposed and unexposed Chinese workers in Tianjin, China, 2004.

	Unexposed *n* (%)	Exposed *n* (%)	*p*-Value
Age (years)^a^			
19–32	14 (42)	20 (61)	0.14
33–49	19 (58)	13 (39)	

Abstinence (days)^a^			
≤ 5	16 (48)	19 (58)	0.46
> 5	17 (52)	14 (42)	

BMI (kg/m^2^)^b^			
< 18.5 (underweight)	1 (3)	3 (9)	0.38
18.5–24.9 (normal)	20 (61)	19 (58)	
25–29.9 (overweight)	10 (30)	11 (33)	
≥ 30 (obese)	2 (6)	0 (0)	

Current tea drinker^b^			
No	29 (88)	23 (70)	0.07
Yes	4 (12)	10 (30)	

Current cola drinker^b^			
No	29 (88)	26 (79)	0.32
Yes	4 (12)	7 (21)	

Chronic disease^b^,^c^			
No	22 (67)	29 (88)	0.04
Yes	11 (33)	4 (12)	

Education^a^			
Completed middle school or less	17 (52)	28 (85)	0.004
Completed high school or more	16 (48)	5 (15)	

Smoked last 3 months^a^			
No	9 (27)	8 (24)	0.78
Yes	24 (73)	25 (76)	

Drank alcohol last 3 months^b^			
No	2 (6)	6 (18)	0.26
Yes	31 (94)	27 (82)	

Hot baths last 3 months^a^			
No	21 (64)	12 (36)	0.03
Yes	12 (36)	21 (64)	

Biked ≥ 0.5 hr/day^a^			
No	16 (48)	11 (33)	0.21
Yes	17 (52)	22 (67)	

Ate fruit and vegetables > 3.6 times/day^a^			
No	22 (67)	15 (45)	0.08
Yes	11 (33)	18 (55)	

a Analyzed by chi-square test.

b Analyzed by Fisher’s exact test.

c Includes self-reported history of high blood pressure, other diseases of the heart or blood vessels, tuberculosis, lung disease, anemia, other blood diseases, diabetes, thyroid diseases, other hormonal diseases, stomach ulcers or other diseases of the gastrointestinal tract, hepatitis, liver disease, epilepsy or other neurologic disorders, or other chronic diseases.

**Table 2:** Summary of three benzene exposure measurements^a^ for benzene-exposed and unexposed workers.

				Percentile					
Measurement	*n*	GM (GSD)	Min	10th	25th	50th	75th	90th	Max
Air benzene (ppm)									
Unexposed	33	—	< LOD	< LOD	< LOD	< LOD	< LOD	< LOD	< LOD
Exposed^b^	33	2.7 (3.9)	< LOD	0.6	1.0	2.9	7.0	18.5	23.6
Low exposed^b^	17	1.0 (2.6)	< LOD	< LOD	0.7	1.0	2.0	4.1	4.6
High exposed	16	7.6 (2.3)	1.4	2.9	4.5	7.7	15.1	22.9	23.6
Total^b^	66	0.6 (5.8)	< LOD	< LOD	< LOD	< LOD	2.9	10.8	23.6

Urinary benzene (μg/L)									
Unexposed	33	0.1 (1.8)	0.1	0.1	0.1	0.1	0.2	0.3	0.9
Exposed	33	14.0 (5.0)	0.8	2.4	4.3	10.3	49.9	117.9	617.0
Low exposed	17	4.2 (2.5)	0.8	1.1	2.4	4.3	7.2	10.3	49.9
High exposed	16	50.0 (3.1)	8.6	11.7	21.1	52.5	116.4	130.9	617.0
Total	66	1.4 (13.3)	0.1	0.1	0.1	0.9	10.3	62.0	617.0

Urinary E,E-MA (mg/L)									
Unexposed^c^	0	—	—	—	—	—	—	—	—
Exposed	33	5.3 (3.4)	0.8	1.1	1.9	6.7	14.4	26.6	40.9
Low exposed	17	1.9 (1.9)	0.8	0.8	1.2	1.9	2.7	6.5	6.7
High exposed	16	16.1 (1.6)	8.3	8.7	11.4	14.4	25.2	28.0	40.9
Total	33	5.3 (3.4)	0.8	1.1	1.9	6.7	14.4	26.6	40.9

Abbreviations: Max, maximum; Min, minimum.

a Urine samples and personal air measurements were obtained from each man at two time points approximately 1 month apart; the GMs of the concentrations among men were used to calculate summary statistics.

b To estimate the GM and GSD, values < LOD were imputed as LOD divided by the square root of 2.

c E,E-MA was not measured in the unexposed group.

**Table 3:** Median and mean aneuploidy frequencies,^a^ and percentage of men with numerical chromosomal abnormalities as determined by XY21 sperm FISH, stratified by benzene exposure group.

	Unexposed (*n*= 33)				Low-exposed (*n*= 17)				High-exposed (*n*= 16)			
	Percent with anomaly^b^	Median	Mean	Range	Percent with anomaly	Median	Mean	Range	Percent with anomaly	Median	Mean	Range
Total hyper- and hypohaploidy	100	13.9	16.2	2.0–41.7	100	13.9	23.5	6.0–100.5	100	19.3	21.7	5.0–50.8
Hyperhaploidy	100	9.9	9.9	2.0–26.9	100	9.0	14.5	2.0–54.0	100	18.4	17.5	4.0–36.8
Disomy X	76	1.0	2.0	0.0–8.0	100	3.0	3.5	1.0–9.0	94	2.0	4.4	0.0–13.9
Disomy Y	88	2.0	2.9	0.0–10.9	82	2.0	3.6	0.0–16.9	94	5.0	6.8	0.0–18.9
X-Y-21	88	3.0	3.8	0.0–16.9	88	3.0	5.3	0.0–32.9	94	3.0	5.2	0.0–13.9
Disomy21	58	1.0	1.2	0.0–8.0	65	1.0	2.1	0.0–17.9	69	1.0	1.1	0.0–4.0
X-21-21	48	0.0	0.8	0.0–5.0	41	0.0	1.3	0.0–12.9	44	0.0	0.6	0.0–3.0
Y-21-21	24	0.0	0.5	0.0–5.0	41	0.0	0.8	0.0–5.0	44	0.0	0.5	0.0–2.0
Hypohaploidy	88	4.0	6.2	0.0–17.9	94	4.0	9.0	0.0–46.8	81	3.5	4.2	0.0–13.9
X_	18	0.0	0.2	0.0–1.0	12	0.0	0.1	0.0–1.0	19	0.0	0.4	0.0–3.0
Y_	9	0.0	0.2	0.0–2.0	12	0.0	0.1	0.0–1.0	19	0.0	0.4	0.0–3.0
Sex nullisomy	85	4.0	5.9	0.0–16.9	94	4.0	8.8	0.0–45.8	81	3.0	3.4	0.0–10.9
Diploidy	94	4.0	7.5	0.0–44.8	100	4.0	7.4	0.0–31.9	94	3.0	7.0	0.0–31.8
Other^c^	33	0.0	0.4	0.0–2.0	41	0.0	0.7	0.0–4.0	50	0.5	0.6	0.0–2.0

a Values shown are frequencies per 10,000 sperm counted; 331,900, 170,934, and 160,935 sperm were analyzed among unexposed, low exposed, and high exposed, respectively. Median and mean frequencies include all participants, and men without a detected anomaly were assigned a value of zero.

b Percentage of men with at least one sperm with this defect per 10,000 sperm analyzed.

c All anomalies not detailed above.

**Table 4:** Adjusted^a^ associations between benzene exposure and sperm aneuploidy outcomes in low- and high-exposure groups.^b^

	Low-exposed vs. unexposed		High-exposed vs. unexposed		
	IRR (95% CI)	*p*-Value	IRR (95% CI)	*p*-Value	*p*trend^c^
Total hyper- and hypohaploidy	1.5 (0.9–2.4)	0.09	1.7 (1.1–2.7)	0.03	0.03
Hyperhaploidy	1.6 (1.0–2.4)	0.03	2.3 (1.5–3.6)	< 0.001	< 0.001
Disomy X	2.0 (1.1–3.4)	0.02	2.8 (1.5–4.9)	< 0.001	0.001
Disomy Y	1.1 (0.6–2.1)	0.78	2.6 (1.4–4.8)	< 0.001	0.002
X-Y-21	1.5 (0.8–2.8)	0.22	1.8 (0.9–3.5)	0.09	0.08
Disomy21	2.1 (1.0–4.7)	0.07	1.6 (0.7–4.0)	0.30	0.20
X-21-21	1.9 (0.7–5.0)	0.17	1.4 (0.5–4.1)	0.56	0.43
Y-21-21	2.4 (0.8–7.2)	0.12	2.0 (0.6–7.3)	0.27	0.18
Hypohaploidy	1.3 (0.6–2.6)	0.49	0.8 (0.4–1.6)	0.51	0.61
X_	0.6 (0.1–3.7)	0.55	2.4 (0.5–10.3)	0.26	0.26
Y_	2.7 (0.2–34.6)	0.44	104 (2.3–4,773)	0.02	0.01
Sex nullisomy	1.3 (0.6–2.8)	0.45	0.6 (0.3–1.4)	0.24	0.36
Diploidy	0.9 (0.4–1.8)	0.76	0.9 (0.4–1.8)	0.71	0.70
Other^d^	2.4 (1.0–6.1)	0.06	3.3 (1.1–9.4)	0.03	0.02

a Each model was adjusted for age, smoking in the past 3 months, hot baths in the past 3 months, regular tea drinking, consumption of fruits or vegetables ≥ 3.6 times per day, and history of any chronic disease.

b Statistical models compared each exposure group with the unexposed group.

c A generalized linear model using a three-category exposure variable was used to assess trend.

d All anomalies not detailed above that include sperm with multiple abnormalities such as X-X_.
